# Supplementary material for: Genome-Wide Association Study of Xian Rice Grain Shape and Weight in Different Environments
Source: Plants (Basel). 2023 Jul 4;12(13):2549. doi: 10.3390/plants12132549 (PMC10347298; doi:10.3390/plants12132549)
Supplement: Supplementary file 1 [file plants-12-02549-s001.zip › plants-2430939-supplementary.pdf]

plants

MDPI

Genome-wide association study of xian rice grain shape and weight in different environments

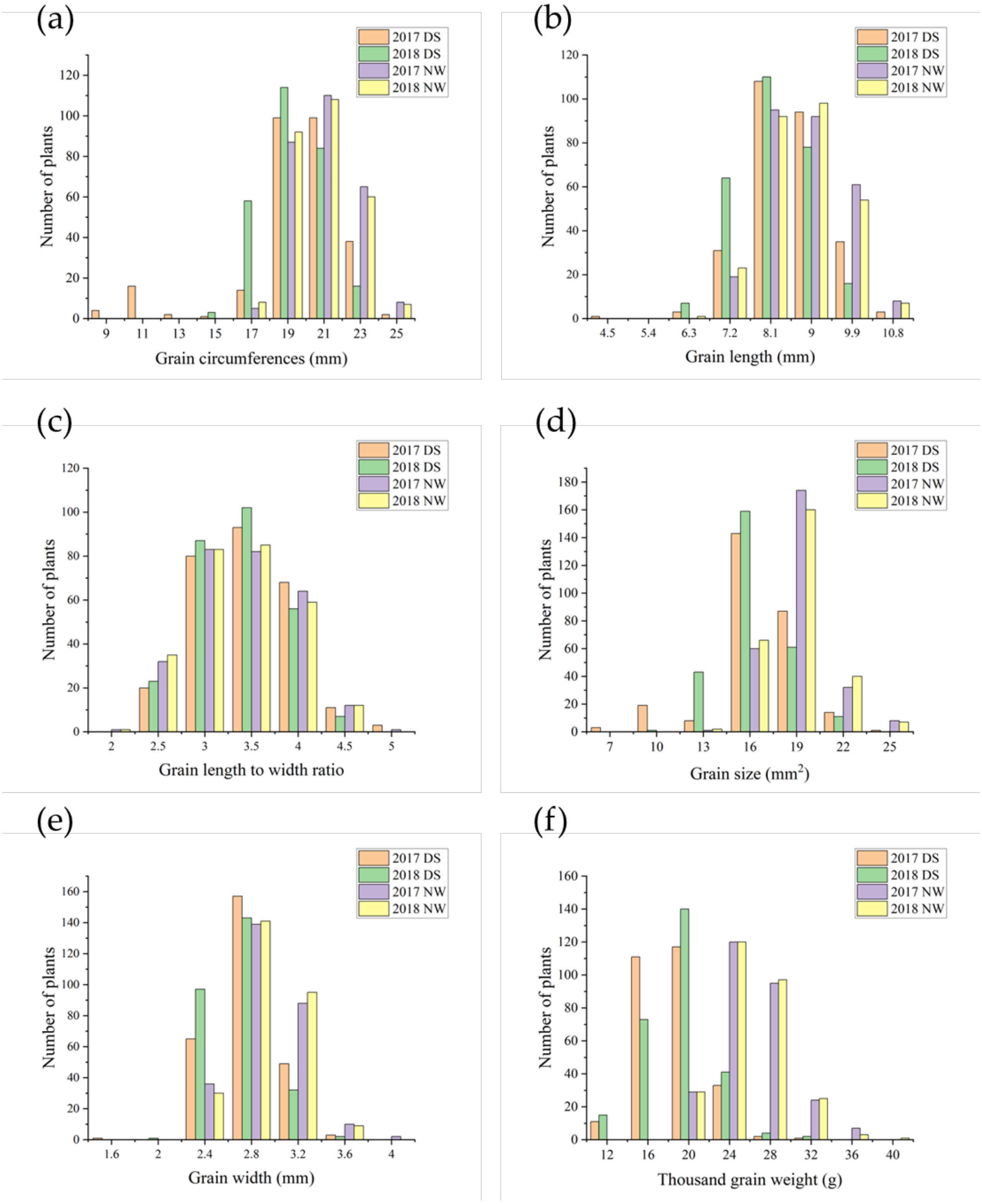

Figure S1. Histogram of the phenotypic frequency distribution of rice grain shape and grain weight in 275 rice accessions. (**a**) Grain circumference;(**b**) Grain length; (**c**) Grain length to width ratio; (**d**) Grain size; (**e**)grain width; (**f**)Thousand grain weight.

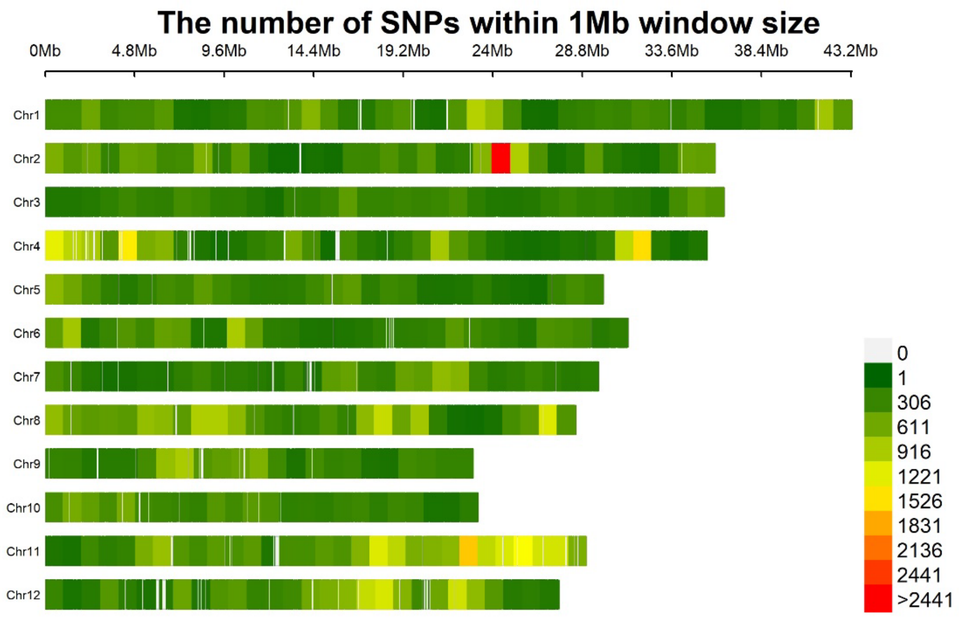

Figure S2. Distribution of single nucleotide polymorphisms (SNPs) and nucleotide diversity across the rice Nipponbare genome in the rice association panel.
